# The effect of neural synchronization on information transmission

**DOI:** 10.1186/1471-2202-14-S1-P366

**Published:** 2013-07-08

**Authors:** Rajeev V Rikhye, Jean-Jacques E Slotine

**Affiliations:** 1 Department of Brain and Cognitive Sciences, Massachusetts Institute of Technology, Cambridge, MA 02139, USA; 2 Nonlinear Systems Laboratory, Department of Mechanical Engineering, Massachusetts Institute of Technology, Cambridge, MA 02139, USA

## 

Networks in the neocortex serve an important purpose of extracting information from sensory inputs. One appealing theory is the efficient coding hypothesis, which postulates that information about a stimulus is maximized when redundancy between neurons is reduced [1]. This gives rise to the notion of a sparse population code where only neurons that are highly selective to certain stimulus features respond. However, sparse codes are highly sensitivity to noise. An alternative strategy is to use a synchronized code, which is robust to noise but reduces the representational capacity of the network [2,3]. Hence, it is only logical to ask what level of redundancy between neurons maximizes the amount of information contained in a population code.

In this study, we investigated how the collective dynamics of a population of neurons represent stimulus-specific information. Our aim was to investigate (1) the conditions required for a network to synchronize and (2) the amount of information contained in a synchronized rate code. By applying nonlinear contraction analysis on a recurrent network with time varying inputs, we found that the degree of synchronization depended critically on the gain of the feedforward projection. Our analysis showed that changing the feedforward gain exploits different symmetries in the network, resulting in different patterns of synchrony and different population codes. We hypothesized that the coding strategy can by optimized by altering this synaptic gain.

We tested this prediction computationally using a two-layered network. The receiver layer was a balanced recurrent network of 5000 integrate-and-fire neurons. Input to the network was provided by a population of "visually responsive" neurons, which was modeled using a linear-nonlinear Poisson (LNP) cascade. The LNP neurons were tuned to 16 orientations and projected nonspecifically to 20% of the neurons in the receiver layer. We assumed that the stimulus was a sequence of drifting gratings with random orientations. In response to stimuli, the network displayed transiently synchronized responses. Because similarly tuned LNP neurons projected to different subsets of neurons, the pattern of network activity was different for each stimulus sequence. By gradually modifying the feedforward gain, we were able to alter this pattern of stimulus-specific synchronization, confirming the results from our mathematical analysis. In the high gain condition, neurons became redundant and responded to all stimuli. However, when gain was low, only sparse subsets of neurons were activated by different stimuli. Finally, we used an optimal linear decoder to measure of how well the network encoded the stimuli. We found that classification accuracy varied as a function of the degree of synchronization between neurons. Classification accuracy was highest when approximately 15% of the neurons responded concurrently to the stimulus. In both densely synchronized and sparse codes, the decoder performed below chance.

Our results show that stimulus-specific information is maximized only when small ensembles of neurons are synchronized. Our finding that stimulus-dependent synchronization is controlled by the gain of feedforward synapses is unique as it suggests that mechanisms which alter synaptic gain such as attention can alter the coding strategy employed by the neocortex.
